# Spatio-Temporal Modelling of Dust Transport over Surface Mining Areas and Neighbouring Residential Zones

**DOI:** 10.3390/s8063830

**Published:** 2008-06-06

**Authors:** Lubos Matejicek, Zbynek Janour, Ludek Benes, Tomas Bodnar, Eva Gulikova

**Affiliations:** 1 Institute for Environmental Studies, Faculty of Science, Charles University in Prague, Benatska 2, Prague 2, CZ 12801, Czech Republic; 2 Institute of Thermomechanics, Academy of Sciences, Dolejskova 5, Prague 8, CZ 18200, Czech Republic; E-mail: janour@it.cas.cz; 3 Department of Technical Mathematics, Czech Technical University in Prague, Karlovo Namesti 13, CZ 12135, Czech Republic; E-mails: benes@marian.fsik.cvut.cz; bodnar@marian.fsik.cvut.cz; 4 Ecoprogress, Zatecka 1899, Most, CZ 43401, Czech Republic; E-mail: gulikova@ecoprogress.cz

**Keywords:** 3D laser scanning, GPS, Spatio-temporal modelling, Dust transport, GIS, Spatial database, Visualization, RANS equations

## Abstract

Projects focusing on spatio-temporal modelling of the living environment need to manage a wide range of terrain measurements, existing spatial data, time series, results of spatial analysis and inputs/outputs from numerical simulations. Thus, GISs are often used to manage data from remote sensors, to provide advanced spatial analysis and to integrate numerical models. In order to demonstrate the integration of spatial data, time series and methods in the framework of the GIS, we present a case study focused on the modelling of dust transport over a surface coal mining area, exploring spatial data from 3D laser scanners, GPS measurements, aerial images, time series of meteorological observations, inputs/outputs form numerical models and existing geographic resources. To achieve this, digital terrain models, layers including GPS thematic mapping, and scenes with simulation of wind flows are created to visualize and interpret coal dust transport over the mine area and a neighbouring residential zone. A temporary coal storage and sorting site, located near the residential zone, is one of the dominant sources of emissions. Using numerical simulations, the possible effects of wind flows are observed over the surface, modified by natural objects and man-made obstacles. The coal dust drifts with the wind in the direction of the residential zone and is partially deposited in this area. The simultaneous display of the digital map layers together with the location of the dominant emission source, wind flows and protected areas enables a risk assessment of the dust deposition in the area of interest to be performed. In order to obtain a more accurate simulation of wind flows over the temporary storage and sorting site, 3D laser scanning and GPS thematic mapping are used to create a more detailed digital terrain model. Thus, visualization of wind flows over the area of interest combined with 3D map layers enables the exploration of the processes of coal dust deposition at a local scale. In general, this project could be used as a template for dust-transport modelling which couples spatial data focused on the construction of digital terrain models and thematic mapping with data generated by numerical simulations based on Reynolds averaged Navier-Stokes equations.

## 1. Introduction and the general approach

The impacts of mineral dust on the natural environment in the neighbourhood of surface coal mines have long been recognized. One prerequisite for estimating these impacts is a determination of wind flows over the precisely mapped surface. Despite existing studies on the subject [1-3], practical numerical calculations have not been possible until recently. This is because the processes governing emissions and transport by wind flows over a surface are very complex. There is a dependency on terrain morphology which causes difficulties in estimating the dust emission rate and determining dust transport. In addition to wind flows, a set of physical processes in soils predetermines the conditions of primary dust dispersion. These processes are affected by highly variable natural factors, such as climate, soil state, and surface roughness. In cases of coal mine surfaces, other factors include mining activities, especially temporary storage, coal sorting, excavators, conveyors, and moving vehicles. The transport of coal dust is characterized by turbulent interaction in the atmospheric boundary layers. The dust particles are often driven by intensive meso-scale to synoptic-scale systems over long distances. At the more local spatial scale of the surface coal mine and neighbouring areas, a more precise spatio-temporal modelling of the mine surface and wind flows are needed for better predictions of coal dust transport.

In order to achieve this spatio-temporal modelling of dust transport over the surface of a mining area and neighbouring residential zones, a geographic information system (GIS) is used to create spatial data structures and modelling tools. Then, numerical simulations of the wind flows supported by a digital terrain model (DTM) can be carried out more precisely. In order to carry out this advanced spatial analysis, recent GIS are used to manage complex data structures and to create spatial models. Finally, visualization methods assist in the creating of map compositions for the presentation of modelling results. Running of GIS on advanced computer systems allows the implementation of these types of time consuming numerical algorithms focused on environmental research [4, 5]. The GIS development tools can assist in creating domain-specific components dedicated to data exchange or numerical modelling.

This paper describes GIS methods for the construction of a DTM and visualization of dust dispersion based on numerical modelling. In order to provide spatio-temporal modelling, a GIS database is created to manage data from 3D surface laser scanners, GPS measurements, existing thematic maps, numerical models and meteorological stations. The system is then applied to a simulation of dust transport over part of a surface coal mine located in the Czech Republic.

## 2. The main data sources for the spatio-temporal modelling

Spatio-temporal modelling is generally considered as a synthesis of GISs, which describe the spatial environment, and environmental models, which are linked to dynamic processes in the natural environment. Environmental models require data about the environment within which the processes occur, and simulations of environmental models provide additional data to extend the environmental descriptions. Thus, GIS can be used to serve as a common analysis framework for environmental models. Originally, GIS and environmental modelling were developed separately, which is indicated by their different data structures and functionality for data exchange and the user interface. Nowadays, however, there are some strategies for how to integrate environmental modelling in the GIS environment. They range from simple data exchange through shared data files to developing analytical tools into fully functional GISs [6]. In the case of spatio-temporal modelling of dust transport, the whole range of methods and functions can be solved using existing GIS capabilities, but extensions are necessary to process the numeric simulations of wind flows.

The main spatial data input for wind flow simulation is a DTM that is based on 3D surface laser scanning, GPS measurements and existing thematic mapping. The specific selection of spatial data inputs for the DTM and visualization was carried out depending on the required precision and actual availability.

## 2.1. Data sources from surface laser scanning

**Figure 1.**
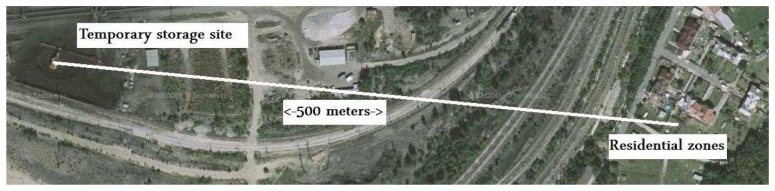
The local area for 3D surface laser scanning (based on the aerial images: Mapy.cz).

**Figure 2.**
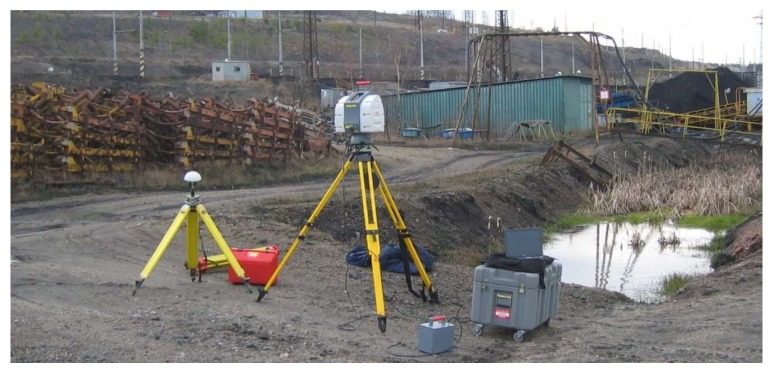
The 3D laser scanner Leica HDS3000 making point cloud acquisition for a slope area on the left, and for the temporary storage place on the right.

3D laser scanners provided an efficient method for local 3D point cloud acquisition of the surface coal mine and its associated industrial installations. Complete coverage with point cloud data mostly requires data acquisition from multiple standpoints. In order to improve existing thematic spatial data, four scans with an average grid size of 0.2 meters were performed with a Leica HDS 3000 3D laser scanner. The selected local area of the surface coal mine is shown in Figure 1. The temporary storage site located on the left represents the dominant dust emission source. The residential zone located approximately 500 meters from the temporary storage place is on the right side. The measurement equipment at one of the standpoints is illustrated in Figure 2.

**Figure 3.**
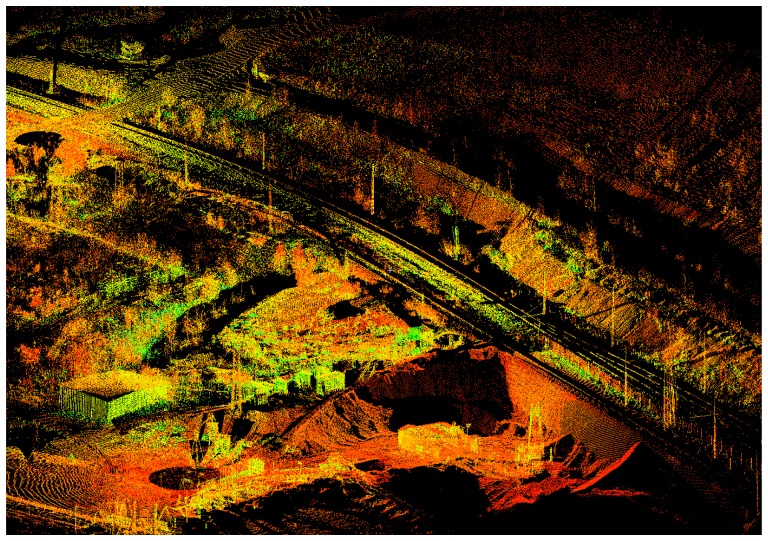
The final 3D point clouds for the local area processed by Leica Cyclone software.

**Figure 4.**
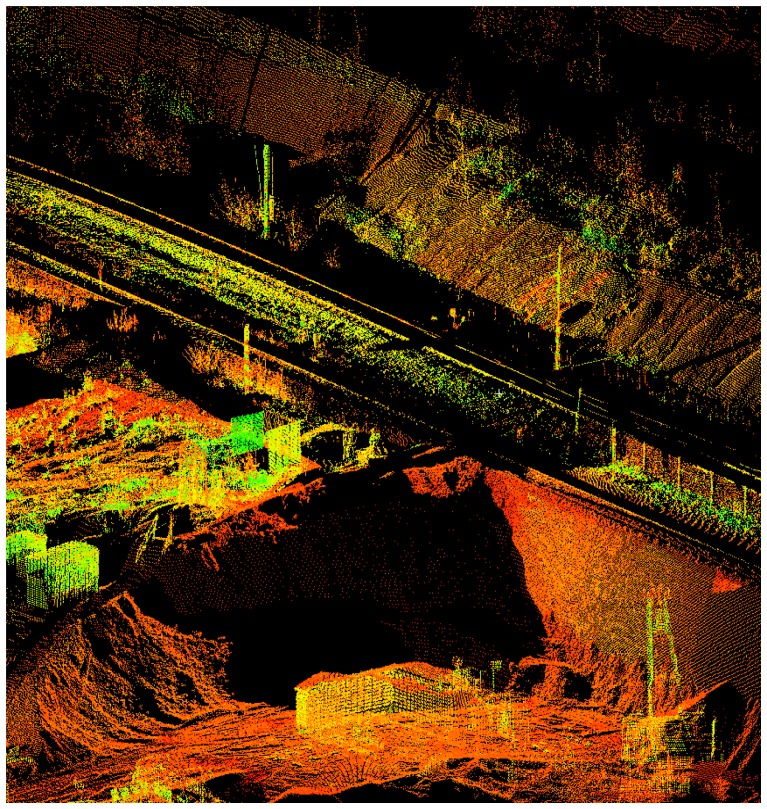
A more detailed view of the temporary storage and sorting place with the coal mine installations and other equipment.

In order to create the required spatial data inputs for such a complex DTM construction [7], processing of the point clouds was performed using Leica Geosystems HDS Cyclone software in cooperation with Gefos Ltd. in the Czech Republic. The final 3D point clouds for the area are shown in Figure 3, and a detailed view of the temporary storage site is in Figure 4.

### 2.2 Data sources from GPS

**Figure 5.**
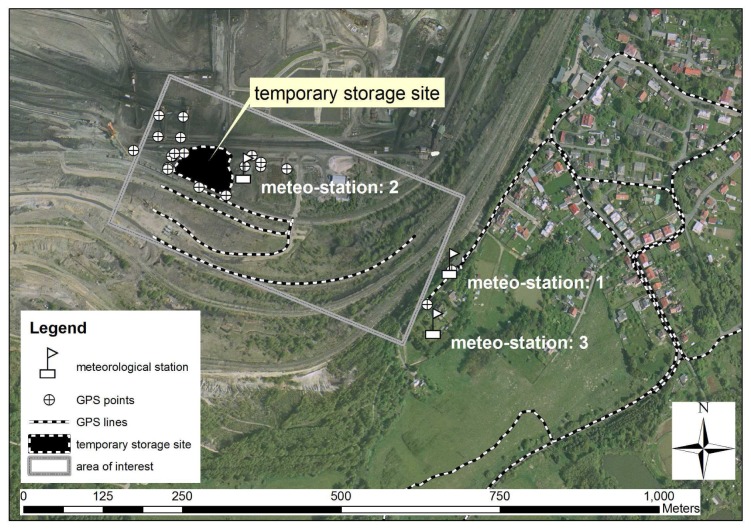
The lines of the surface mine slopes, the boundaries of the temporary storage site and the locations of other surface objects.

**Figure 6.**
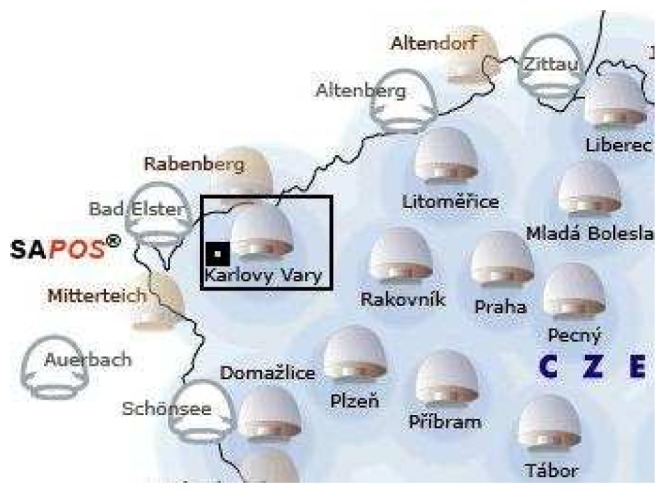
The local network of the GPS reference stations (CZEPOS) in the Czech Republic.

While the 3D surface laser scanning was used for mapping of continuous parts, a GPS Trimble-Pathfinder ProXT was used to assist in the capturing of the lines of the surface mine slopes, the boundaries of the temporary storage site and the locations of other surface objects (transport routes, other potential emission sources and meteorological stations). The basic measurement scheme is shown in Figure 5. The accuracy of the GPS measurements was improved in the postprocessing phase using data from the nearest reference station in the Czech Republic (Karlovy Vary, Figure 6). The final accuracy of the spatial data after the postprocessing phase was 0.5 m.

### 2.3 Data sources from meteorological stations

**Figure 7.**
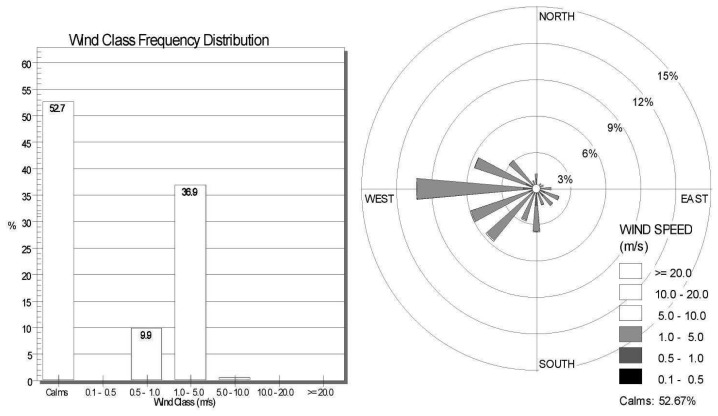
The wind class frequency distribution and the wind rose based on observations from the local meteorological stations (processed by the WRPLOT View software).

Data including wind speed, wind direction and atmospheric pressure consisted of time series measurements taken at local meteorological stations. There are two meteorological stations located in the area of the surface coal mine, and a third station which also includes soil humidity measurements is located in the neighbouring residential zone. The data are archived at regular intervals. A selected time period is shown in Figure 7. The wind rose illustrates the predominant wind directions causing increased dust transport to the residential zone. The data processing is based on observations from all the local meteorological stations, which showed similar results for the selected time period.

### 2.4 Emission sources

Dust from surface mines is emitted into the atmosphere from a wide range of sources that can be classified as either passive emission sources (temporal coal storage sites, eroded slopes and piles) or active emission sources (sorting sites, excavators, transport routes and other mining equipment). The term dust is non-specific with respect to the size, shape and chemical properties of the particles. Dust is mostly formed by turbulent wind action accompanying the release of gaseous emissions. The concentration of particles in the atmosphere can usually range from a few micrograms to hundreds of micrograms per cubic metre. There have been a number of studies assessing the risk of dust as a health issue for mine workers and surrounding natural environments [8].

**Figure 8.**
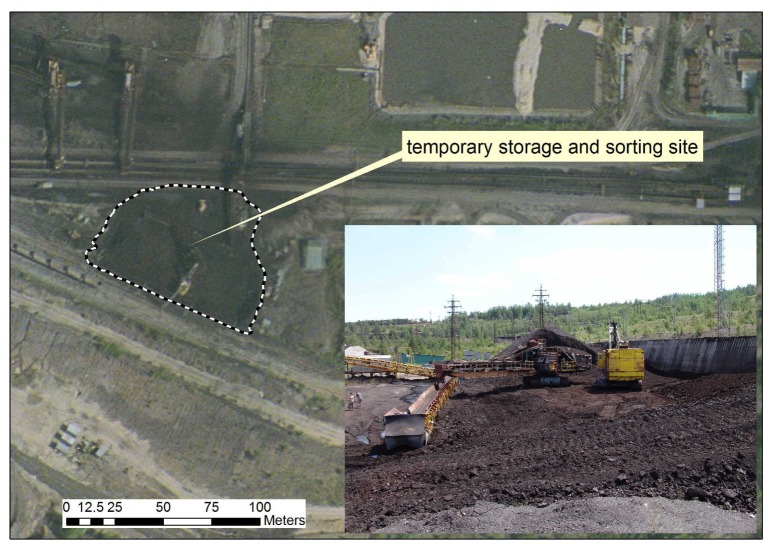
The temporary storage and sorting site located with GPS measurements. The attached image illustrates the actual status of the site.

In this study we focused on one dominant emission source, the temporary coal storage and sorting site, which was identified by the classification of satellite images or aerial images. In order to more precisely determine its location and shape, terrain measurements with a GPS were used to create a map layer in the framework of the GIS project. Figure 8 shows the area around the temporary storage and sorting site, and the inset shows the current status, with mining installations and temporary stockpiles.

### 2.5 Other data sources

Data sources for the DTM are complemented by contour lines from existing digital map sources at a scale of 1:10,000. These maps do not just cover the surface mining area, but extend the DTM to the larger area of the surface mine and its neighbouring zones. Other thematic map sources are Internet based geographic data. For this surface mine, located in the western part of the Czech Republic, Internet mapping servers based on ESRI technology (http://geoprotal.cenia.cz and http://mapy.kr-karlovarsky.cz) were used to add thematic map layers. In addition to the laser scanning, the local area of the temporary storage site was extended by spatial data obtained from a mining company mapping survey (transport routes, locations of the mining installations, and a set of 3D points).

Satellite images can display larger surface emission sources such as whole surface mine regions. In the case of passive optical sensors, the natural energy detected is emitted or reflected by the objects themselves or by the surrounding area being observed. Then, the spectral, spatial and temporal properties of the gathered information are analysed by the remote sensing software tools. In addition to the natural energy of the visible electromagnetic light spectrum, satellites (LANDSAT, ASTER, SPOT) can detect infrared light that human eyes cannot perceive. Bands in the infrared spectral range can be used for the discrimination of geologic rock types, soil boundaries and soil moisture content which affect the rate of surface emission sources. Relationships with known features on the ground (bare land, vegetation, urban, forest) can be found using multispectral classification. In this study, an orthorectified image from LANDSAT 7 ETM+ (192/25, RMS = 0.63 pixel, approximately 19 meters) was used to display the entire area including the surface mine and residential zones in the framework of the visualization phase.

### 2.6 The GIS approach

**Figure 9.**
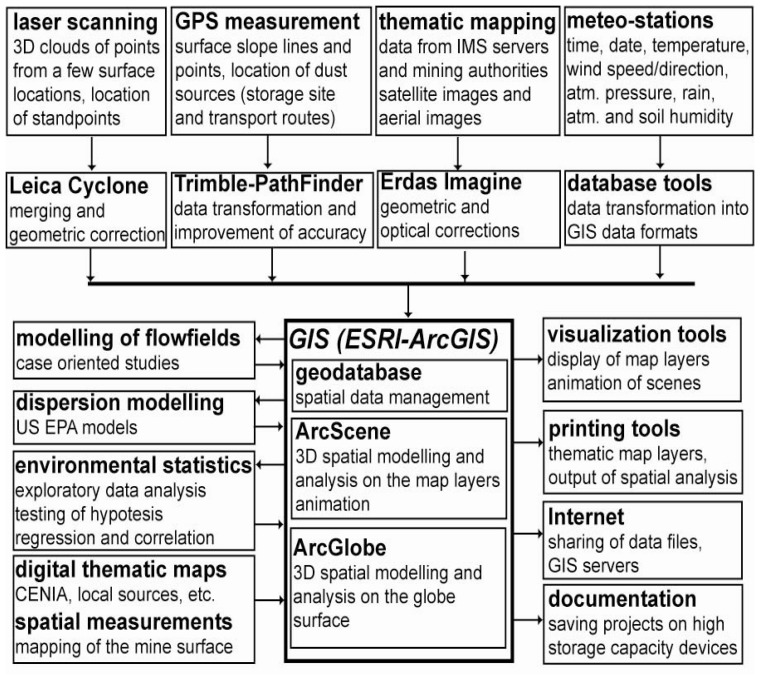
The general workflow of data processing in the GIS environment.

Spatio-temporal modelling including spatial analysis and extensions for the analysis of time series offers a wide range of advanced functions for data processing, visualization and digital mapping outputs [9-11], and thus can support environmental planning and policy making. Direct access to large volumes of data allows more complex information to be used in decision-making processes, while the ability to reach a broad audience and to generate a large number of options, scenario analyses and forecasts can help to more efficiently solve environmental problems [12]. In the case of dust transport, insight into wind flows together with the DTM, aerial images and pre-processed data from meteorological stations and existing thematic map layers helps in the exploration of the mechanisms of dust dispersion and deposition. This approach focused on the integration of spatio-temporal data and the workflow of data processing is illustrated in Figure 9.

The GIS project based on spatio-temporal data serves as the main data storage for numerical modelling. Data inputs for the numerical simulations contain the DTM and time series from the meteorological stations. Data outputs from numerical simulations are represented by the three dimensional domain over the DTM. The domain forms layers, which in turn contain a set of nodes. The attributes of each node include the coordinates in 3D space, the velocity vector, atmospheric pressure and the concentration of the passive pollutant. In order to import data into the GIS project, the post processing phase is focused on georeferencing of the data domain, transformation of the internal data and import into the GIS database.

### 2.7 The modelling approach

The dispersion of dust particles near an emission source can be described by mathematical equations based on physical principles [13]. However, in the actual environment with a huge number of various surface objects (i.e., the temporary storage site, mining installations and buildings) and other factors (climate, soil conditions), the models reflect only basic phenomena and have to be improved through many corrections depending on the selected mining area and field measurements [14, 15].

Due to this approximate character of the results from mathematical modelling, an alternative method is to use wind tunnels for physical modelling. These types of experiments can be performed on down-scaled models of the studied area, with simulated flow conditions similar to the conditions observed in the atmosphere. In order to validate flow fields and pollution dispersion in simplified 2-D and 3-D space, field measurements are necessary to explore all the spatio-temporal characteristics which depend on surface objects [16].

In this paper, wind flows and pollution dispersion in atmospheric boundary layers were simulated through numerical models based on Reynolds averaged Navier-Stokes equations (RANS) in non-conservative form:

(1) $$u_{x}+v_{y}+w_{z}=0$$

(2) $${\text{V}}_{\text{t}}+{\text{uV}}_{\text{x}}+v{\text{V}}_{y}+w{\text{V}}_{z}=-\frac{\nabla p}{\rho }+{\left[{\text{KV}}_{x}\right]}_{x}+{\left[{\text{KV}}_{y}\right]}_{y}+{\left[{\text{KV}}_{z}\right]}_{z}\;,$$

where *V* = *col*(*u*,*v*,*w*) is the velocity vector, *p* pressure, *ρ* density.

The transport equations for concentrations of passive pollutants are:

(3) $$C_{t}^{i}+{\text{uC}}_{x}^{i}+{\text{vC}}_{y}^{i}+{\text{wC}}_{z}^{i}={\left[K\frac{c_{x}^{i}}{\sigma_{c^{i}}}\right]}_{x}+{\left[K\frac{c_{y}^{i}}{\sigma_{c^{i}}}\right]}_{y}+{\left[K\frac{c_{z}^{i}}{\sigma_{c^{i}}}\right]}_{z},$$

where *C^i^* is the concentration of the *i_th_* pollutant and *σ* denotes the turbulent Prandtl's number. The turbulence model is based on the Boussinesq hypothesis of the turbulent diffusion coefficient:

(4) $$K=\nu +\nu_{T}$$

that is represented by the sum of molecular and eddy viscosity. Thus, the final implemented algebraic turbulence model is in the form:

(5) $$\begin{array}{ccc} K=\nu +l^{2}\sqrt{\left[{\left(\frac{\partial u}{\partial z}\right)}^{2}+{\left(\frac{\partial \nu }{\partial z}\right)}^{2}\right]}, & \;\text{where}\;l=\frac{\kappa_{2}\left(z+z_{0}\right)}{1+\kappa_{2}\frac{z+z_{0}}{i_{\infty }}}, & l_{\infty }=\frac{27|V_{G}|10^{-5}}{f_{c}} \end{array}$$

The parameter *f_c_* = 0.00011 *ms* denotes the Coriolis parameter. The parameter *V_G_* is the geostrophic wind. The numerical simulation was carried out with a semi-implicit finite-difference scheme [17] extended by a number of other simulation tools focused on pollution dispersion in 3D atmospheric boundary layers [18].

**Figure 10.**
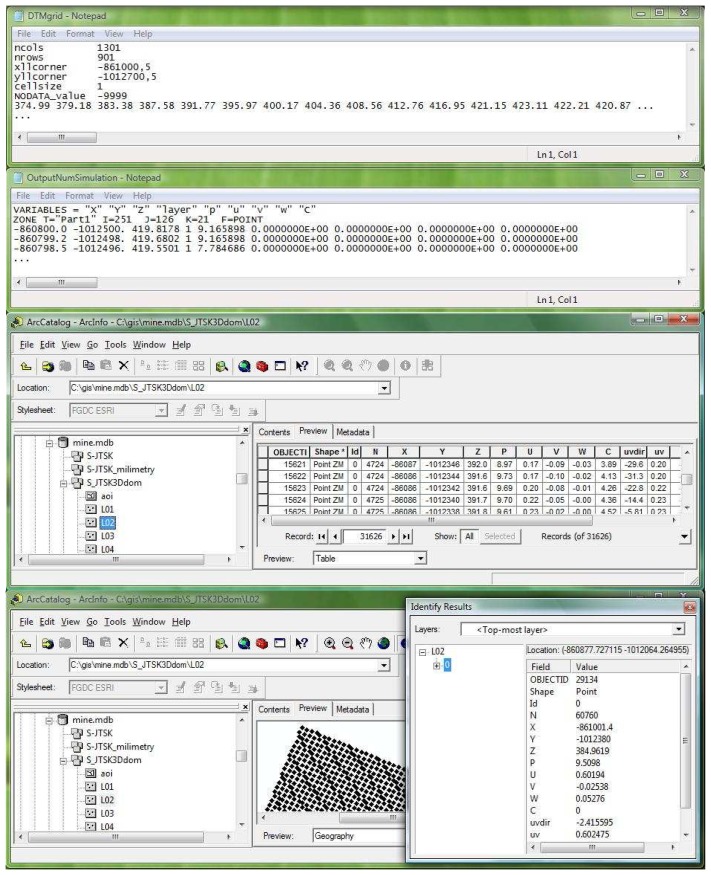
The data exchange by shared files between the GIS and numerical simulation **(a)** An example of the DTM exported into the ASCII grid for numerical simulation. **(b)** An example of the outputs from numerical simulation represented by 3D points (*X*, *Y*, *Z* coordinates) and their attributes: atmospheric pressure (*p*), wind speeds in the *x*, *y*, *z* direction (*u*, *v*, *w*), and dust concentration (*c*). **(c)** An example of the geodatabase (*mine.mdb*) in ArcGIS containing selection of the point layer with the attribute table based on data from the numerical simulation (illustrated on the left). **(d)** An example of the geodatabase (*mine.mdb*) in ArcGIS containing selection of a point.

The outputs from numerical simulations were formed in the 3D domain and divided into layers with a set of spatial points. These layers are located over the area of interest. An example of data exchange by shared files is illustrated in Figure 10. Initially, the DTM was exported into an ASCII grid as the input file for the numerical simulation of wind flows and pollutant concentrations (cell size: 1 meter; national coordinate system in the Czech Republic: S-JTSK). The outputs from numerical modelling were then backward imported into the GIS database. The text file contained 3D points (*X*, *Y*, *Z* coordinates) and their attributes: atmospheric pressure (*p*), wind speeds in the *x*, *y* and *z* direction (*u*, *v*, *w*), and dust concentration (*c*). Finally, the GIS database (ESRI's geodatabase in ArcGIS) contained 21 point layers from the imported 3D domain located over the area of interest. The outputs from numerical simulation (values of variables: *p*, *u*, *v*, *w* and *c*) were then transferred into attribute tables for subsequent processing and visualization.

### 2.8 Visualization of the dust transport

**Figure 11.**
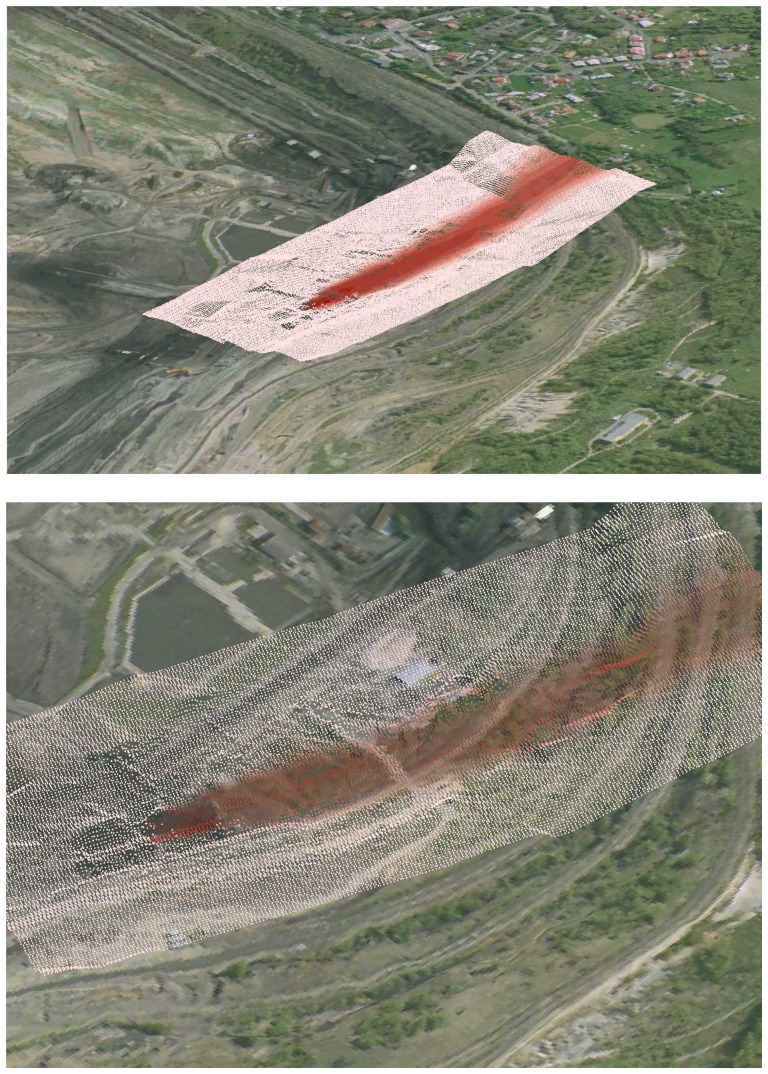
(**a**) An example of the visualization of dust concentration in the selected horizontal layer 5 meters over the DTM (extended by the simulation grid and exported from ArcScene). **(b)** A more detailed view of the visualization.

There are various methods for visualization that depend on available computer simulation tools. Existing visualization tools are mostly included with simulation packages as extensions or programming libraries [19]. Some visualization tools are included directly into the user interface [20]. Many methods are implemented in the GISs that can usually employ a two-dimensional framework included into the map layers. But, wind flows and dust transport require a more dynamic way of data processing and visualization, because the processes vary through three-dimensional space and through time. In spite of the fact that conventional GISs can handle the third dimension and time as attributes of basic geographical objects, other standalone software applications are needed to offer more advanced visualization functions. In many cases, existing GISs are extended by modules that provide visualization toolkits for 3D spatial data and time-varying data [21]. In this instance, ArcScene in ArcGIS was used for the visualization of atmospheric pressure, wind flows and dust concentrations over the area of interest. As an example, Figure 11 illustrates the 3D visualization of the dust concentration in one selected layer. The image is based on spatial data exported into the graphic files that allow only a limited demonstration of full functionality.

**Figure 12.**
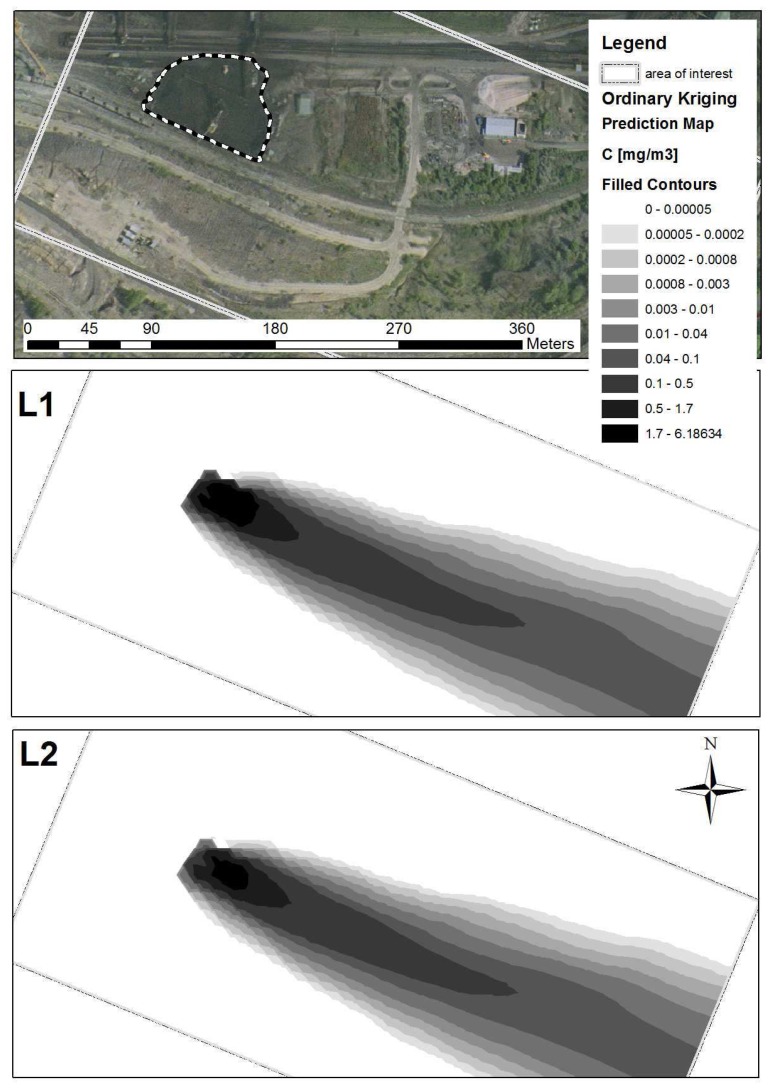
An aerial image of the area of interest followed by the two bottom layers (L1, L2) with the prediction maps of dust concentration. The prediction maps are based on spatial interpolation (ordinary kriging) of the data transferred from the numerical simulation.

**Figure 13.**
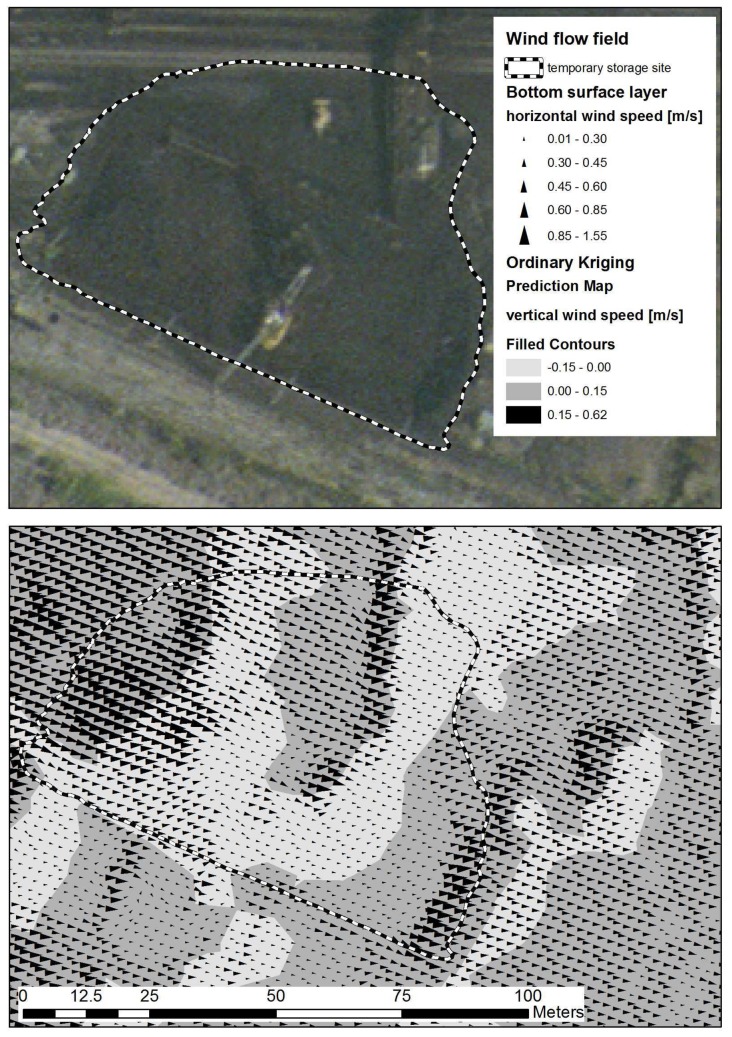
An aerial image of the temporary storage site complemented by the wind flows in the first bottom layer over the storage site (approximately 1 meter over the stockpiles). The arrows show the horizontal speed and direction; the filled line contours on the background show the vertical speed.

The 3D domain transferred into the GIS contains 21 layers with a height increment of 1 meter for the 15 bottom layers, and a height increment of 100 meters for the 6 upper layers. For example, the 2 bottom layers with the prediction map of dust concentration over the area of interest are shown in Figure 12. The first bottom layer (L1) is 1 meter over the DTM, and the second layer (L2) is 2 meters over the DTM. In order to study wind erosion processes over the coal stockpiles in a more precise way, Figure 13 illustrates a detailed view of the first bottom layer over the temporary storage and sorting site. The arrows show the wind direction and wind speed over the stockpiles. Vertical drift is illustrated by the prediction map in the background.

### 3. Discussion and suggested future research

This study describes the extension of the capabilities for spatio-temporal analysis, with an emphasis on physically based dust transport and coupling with GIS. This more precise numerical simulation of dust transport requires a more complex DTM construction. This DTM is extended by additional surface objects, some spatial data sources and field measurements, which are then used for construction in the GIS environment. At the same time, various results can be compared depending on accuracy and capture efficiency.

The use of 3D laser scanners enables more accurate continuous mapping of smaller areas for the DTM, but multiple scans from different locations are required to obtain complete data sources from parts that are obscured. Also, more powerful software tools are needed to bring the clouds of spatial points into a common reference system and to eliminate incorrect spatial points, originating for example from vegetation, pylons and mining equipment. In general, a reduced data set is first used for integration into the GIS database in order to create the DTM. Then, the DTM based on point clouds captured with average grid 0.2 meters are a necessary spatial data source for representing the vertical slopes of the surface mines.

GPS measurements with a final accuracy of 0.5 m after the postprocessing phase complement the data from 3D laser scanners. Besides capturing other 3D points and 3D lines for the DTM, the GPS was used for the localization of other surface objects for thematic mapping (i.e. boundaries of the temporary storage site with its coal stockpiles, actual transport routes, coordinates of meteorological stations), and also to complement existing thematic maps of the neighbouring residential zones.

Other 3D spatial data, mostly based on 1:10,000 thematic maps, are not used for the DTM which supports the numerical simulations, but are dedicated to the overall visualization of larger areas extended by data from numerical modelling and spatial analyses. Also, the aerial images and satellite images from Landsat 7 ETM+ are used to more realistically display the area of interest.

Pre-processed data from the DTM and time series of the wind speed and direction from the local meteorological stations are used as data inputs into the numerical simulation based on RANS equations. The simulation output represents a 3D domain of nodes with defined velocity vectors, atmospheric pressure values and dust concentrations. The spatial projection of the 3D domain over the DTM enables the identification of areas of higher wind speed with vertical drift near the stockpiles, where dust emissions are most likely to occur. In addition to the spatial linkage of the simulation outputs with the DTM, this integration of thematic map layers and aerial images in the GIS environment allows new ways to interpret results. Thus, more precise terrain mapping of the surface by GPS and classification of the aerial images can help to improve estimates of dust emission and the modelling of dust transport. Until recently, a number of studies focused on flows over simplified objects such as stockpiles or man-made barriers have been carried out using numerical modelling or simulations in wind tunnels [22, 23]. However, it is difficult to apply these partial results in the general scale of wind flow spatio-temporal modelling over the DTM in real conditions. Thus, to improve model accuracy, more complex measurements of emission processes which depend on meteorological conditions and soil properties are needed to test other effects and to propose more efficient procedures for decreasing dust emissions and deposition.

In order to implement spatio-temporal modelling in the framework of dust transport risk assessment, a standalone application in the GIS environment needs to be developed that can share a wide range of existing spatial data and provide on-line numerical simulations, spatial analysis of dust concentrations and final visualization. Various case studies could then be tested by mining authorities and local agencies, in order to minimize the actual coal emissions and transport.

## 4. Conclusions

The presented spatio-temporal modelling of dust transport over a surface mining area and neighbouring residential zones offers an excellent opportunity for an exploration into dust emission, transport and deposition coupled with data from remote sensors like 3D laser scanning and aerial images. The created GIS project is able to analyse a wide range of data in order to assist in decision-making processes for decreasing dust deposition in residential zones. Various configurations of man-made barriers as well as different flow conditions can be tested to find the most appropriate solutions.
